# Quality analysis of smart phone sleep apps in China: can apps be used to conveniently screen for obstructive sleep apnea at home?

**DOI:** 10.1186/s12911-019-0916-7

**Published:** 2019-11-15

**Authors:** Zhao-feng Xu, Xin Luo, Jianbo Shi, Yinyan Lai

**Affiliations:** Department of Otorhinolaryngology, The First Affiliated Hospital of Sun Yat-sen University, Sun Yat-sen University, No. 58 Zhongshan Second Road, Yuexiu District, Guangzhou, 510080 People’s Republic of China

**Keywords:** Sleep app, Smartphone, Obstructive sleep apnea, China

## Abstract

**Background:**

Obstructive sleep apnea (OSA) is a sleep disorder with a high prevalence in China. Standard diagnosis of OSA requires polysomnography (PSG). Currently, smart phone applications (apps) are widely used as an important source of health guidance. However, the quality of the information provided by these apps has not been carefully assessed.

**Methods:**

We searched for sleep apps available in China. We designed an evaluation scale that included scientific, functionality and usability, and accountability domains. The Scientific domain included an index of 16 items to evaluate the scientific quality of the apps for their level of adherence to PSG. The functionality and usability domain included 10 items to evaluate the functions of apps and 1 item to define whether the apps needed to connect with other devices. The accountability domain included 9 items that came from the Silberg Scale to evaluate whether the information provided by apps were trustable or not. We then calculated the sum of all domains. We also evaluated the popularity of each app.

**Results:**

A total of 2379 apps were found, and 127 met the inclusion criteria. The mean total score of the apps was 14.23 ± 3.93. The mean scores of scientific basis, functionality and usability, and accountability were 5.51 ± 2.58, 2.90 ± 1.84, and 2.90 ± 1.84. The scientific scores of apps that could connect to other devices were higher than those of apps that worked alone (mean score: 5.26 vs. 4.17, *P* < 0.001). The functionality and usability score was correlated with the accountability score, and the coefficient of correlation was 0.304 (*P* = 0.001).

**Conclusions:**

Apps that could connect to other devices were more scientific and powerful than those that worked alone. Multifunctional apps were more popular and reliable. Because of the low quality of sleep apps in China, more work is necessary to create an ideal app.

## Introduction

Obstructive sleep apnea (OSA) is characterized by repetitive episodes of upper airway obstruction occurring during sleep. Snoring, daytime sleepiness, insomnia, and poor sleep quality are common among people who suffer from OSA [1]. According to an epidemiological survey in Guangxi, China, the prevalence of OSA was 4.1% [2]. OSA may lead to hypertension, an increased risk of coronary heart disease, and certain psychosomatic diseases [3, 4].

The standard diagnosis of OSA requires the presence of typical clinical symptoms, such as snoring and daytime sleepiness, and polysomnography (PSG) results [5]. However, polysomnography needs to be performed in a hospital by specially trained doctors and nurses and is not available at many hospitals. Besides, the cost of polysomnography is quite high. Recently, telemedicine using mobile health applications (apps) has become broadly accepted and has helped with the distribution of limited medical resources.

Mobile phones have become an essential necessity, and smart phone applications (apps) have greatly facilitated tasks in our daily lives. In 2016, there were approximately 660 million people surfing the Internet with mobile phones. There are more than 2.2 million apps available for the iOS system in China and more than 1.6 million apps available for the Android system. Globally, the number of mobile health app downloads in 2017 was approximately 3.7 billion [6]. Apps are poised to become a major source of health guidance, and sleep apps are one of the most popular among all of the mobile health apps [7].

The purpose of this study was to evaluate the quality of sleep apps that can be acquired in China and to assess the capacity of Chinese sleep apps to primarily screen for the diagnosis of OSA. Using the results of the study, our goal was to determine how to develop a science-based and practical app to conveniently screen for OSA.

## Methods

### App selection

We used the words “***sleep***” and “***snoring***” (in Chinese) to search for apps in the iOS App Store and the 360 Android Store. The inclusion criteria were 1) apps that had the function of sleep monitoring (i.e., the app can record the sleep duration or track movement and heart rate during sleep) and 2) were displayed in simplified Chinese characters or had simplified Chinese characters available. Apps were excluded if they were not available in mainland China or were not accessible because of broken links. Each app underwent an initial screening based on the description in the app store. Additional data were collected from the developer’s website. All apps that satisfied the inclusion criteria were downloaded and analyzed in May 2018 in Guangzhou, China. For apps available in both the iOS App Store and the 360 Android Store, we used the apps in the iOS Store for data abstraction.

### App evaluation scale

Based on previous studies and tools used to evaluate the quality of online health information, we designed an evaluation tool to analyze the apps (Table 1). The evaluation tool included 3 domains: Scientific Basis, Functionality and Usability, and Accountability [8].

**Table 1:** Evaluation Scale of Apps

Evaluation Scale	
Scientific Basis	
1) Electroencephalography (EEG)	
2) Electrooculography (EOG)	
3) Electrocardiography (ECG)	
4) Chin Electromyography	
5) Nasal-oral airflow	
6) Chest and abdominal piezoelectric band 7) Pulse oximetry	
8) Actigraphy	
9) Anterior tibialis electromyography	
10) Sleep report including sleep structure	
11) Sleep report including Sleep stages	
12) Oxygen saturation	
13) Cardiac events	
14) Respiratory events	
15) Questionnaires to evaluate clinical symptoms	
16) Other complication (e.g., hypertension, cognitive dysfunction, type 2 diabetes and others)	
Functionality and Usability	
1) Smart Alarm clock	
2) Sleep Knowledge	
3) Sleep Aid	
4) Sleep Diary	
5) Personal Information	
6) Communication Platform	
7) Consulting the doctors	
8) Fitness tracking	
9) Blood Pressure Monitoring	
10) Snoring monitoring	
11) Whether the app required other equipment to monitor sleep condition	
Accountability	
1) Authors credited	
2) Authors’ affiliations	
3) Authors’ credentials	
4) Information sources given	
5) References given	
6) App ownership disclosed	
7) Sponsorship disclosed	
8) Application modified in the previous month	
9) Creation or last modification date specified	

### Scientific basis

According to the third edition of the International Classification of Sleep Disorders, the diagnostic criteria of OSA includes an obstructive respiratory disturbance index (RDI) score of ≥5 events/h, as monitored by PSG, and typical clinical symptoms such as daytime sleepiness, loud snoring, witness apnea, episodes of gasping or choking, and body movement that disrupts sleep, or other complications (e.g., hypertension, cognitive dysfunction). Patients with an obstructive RDI ≥ 15 events/h in the absence of clinical symptoms also satisfy the diagnostic criteria of OSA [1].

To evaluate daytime sleepiness, there are several clinical tools, such as the Berlin questionnaires (BQ), Epworth Sleepiness Scale (ESS), and Stop-Bang questionnaires [9]. These questionnaires are usually used to initially screen for sleep apnea and play a role in the diagnosis of OSA.

To evaluate the sleep quality, the standard PSG should include electroencephalography (EEG), electrooculography (EOG), electrocardiograph (ECG), chin electromyography, nasal-oral airflow, a chest and abdominal piezoelectric band, pulse oximetry, actigraphy, and anterior tibialis electromyography [4]. PSG reports should contain data regarding sleep structure, sleep stages, respiratory events, oxygen saturation, and cardiac events.

Thus, the Scientific Basis domain included an index of 16 items to evaluate the scientific quality of the apps for their level of adherence to PSG administration and results and their capacity to detect typical clinical symptoms of OSA. The items are summarized in Table 1. Each of items was coded as 0, indicating “no information provided”; 1, indicating “part of the information provided”; or 2, indicating “correct and complete information”.

### Functionality and usability

Ong et al. [10] evaluated the functionality of 51 selected sleep apps in 2016 and found that in addition to analyzing sleep structure, some apps could also be used as movement trackers, sound recorders, smart alarms and so on. Other studies also described these additional functions of sleep apps [11–13]. Users may download these apps because of their additional functions. All apps need sensors to collect information during sleep times. Some apps used the accelerometer in the mobile phone, while others required other wearable devices or sensors to monitor sleep [14]. Therefore, to evaluate the functionality and usability of these apps, the Functionality and Usability domain was divided into 2 parts. For the Functionality domain, we used functionality evaluation criteria reported in previous studies [10–14] to appraise the functionality of several apps and then adjusted the criteria according to the actual situation. Each item was coded as 0, indicating “does not describe the function”; or 1, indicating “describes the function”.

For the Usability domain, we examined if the app required other equipment to monitor sleep. A score of 0 indicated “the app must connect to other equipment to work properly”, 1 indicated “the app can work independently”, and 2 indicated “the app can work independently and connect to other equipment”.

### Accountability

Accountability was rated on the Silberg scale. The Silberg scale is widely used to judge whether the information is credible, reasonable, or useful [15]. The scale includes 9 questions divided into 4 parts: authorship (authors and contributors, author affiliations, relevant credentials of the authors), attribution (the sources of information, the references of source), disclosure (the ownership of apps were disclosed, the sponsors of app were disclosed) and currency (the apps were modified in the previous months; the date of modification should be specified). In the evaluation of app Accountability, 1 point was awarded for the presence of each of the items, and the maximum score was 9 points.

### Popularity

We also evaluated the popularity of each app based on user rating data and consumer reviews available in the app stores. The user rating scale of apps in the 360 Android store ranges from 0 to 10, while the scale in the iOS store ranges from 0 to 5. To compare the popularity of apps between the 2 scales, we converted the maximum user rating in the 360 Android Store to a score of 5 points.

### Evaluation procedure

All apps were downloaded and coded for each criterion of the evaluation scale: Scientific Basis (16 items, 32 points), Functionality and Usability (11 items, 12 points), and Accountability (9 items, 9 points). Each app was then given a total score (maximum total score for all 3 domains = 53 points). The apps were then ranked based on the total score. The specific scores of each app are shown in Additional file 1: Figure S1. One assessor (XZF) conducted all of the app quality evaluations and calculated the primary evaluation score, and the results were verified by a second assessor (LYY). If there were differences between the two assessors, the results would be discussed by the two assessors and obtain the final evaluation score. SPSS Statistics for Windows, version 13.0, and Excel 2010 for Windows software were used to perform the statistical analyses.

## Results

### App selection

**Fig. 1:**
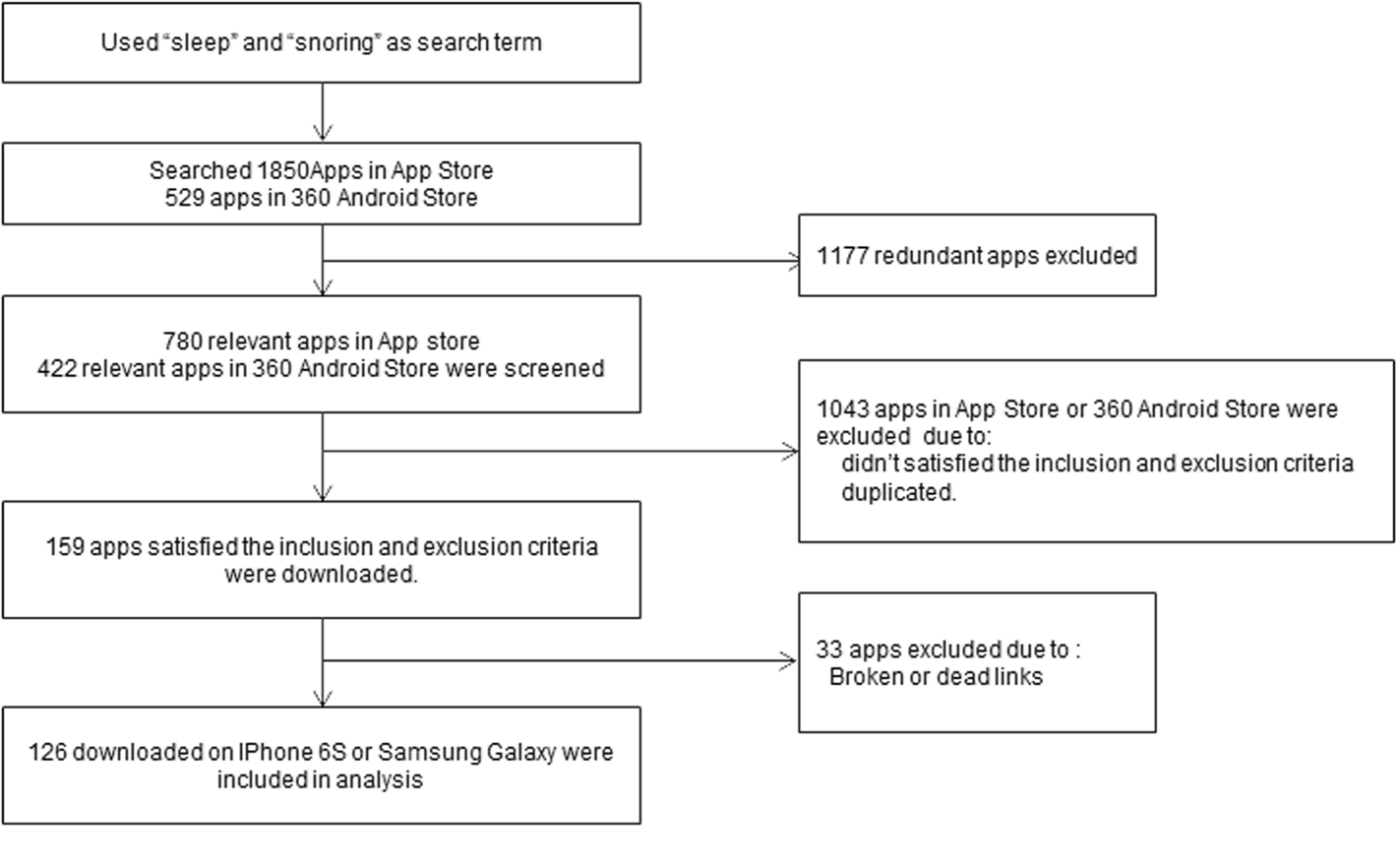
Flow diagram of application selection

A total of 2379 apps (Fig. 1) were found in the 2 app stores (android, *n* = 529; IOS, *n* = 1850). After initial screening based on information provided by the app stores and the developers’ websites, we excluded 1176 apps that were completely irrelevant. The remaining apps were then analyzed based on the inclusion and exclusion criteria. Apps with the same content under different names were excluded (*n* = 1043). Of the remaining 160 apps, 33 were excluded because they could not be downloaded due to broken links or they were not functional. Thus, 127 apps were downloaded and installed on either an iPhone 6S or a Huawei Mate8 for analysis.

### App quality assessment

#### Sample characteristics

All of the 127 apps were consumer sleep apps. The name of each app was derived from Chinese Pinyin or an English translation. The information for each app is presented in Additional file 1: Figure S1.

The overall mean evaluation score of all 127 apps was 14.23 ± 3.93. The app *Taiir SleepCare* received the highest total score of 27, followed by *UmindSleep* and *Sleep as Android* with scores of 26. The app *Sleep Course Recorder* had the lowest total score of 6.

**Fig. 2:**
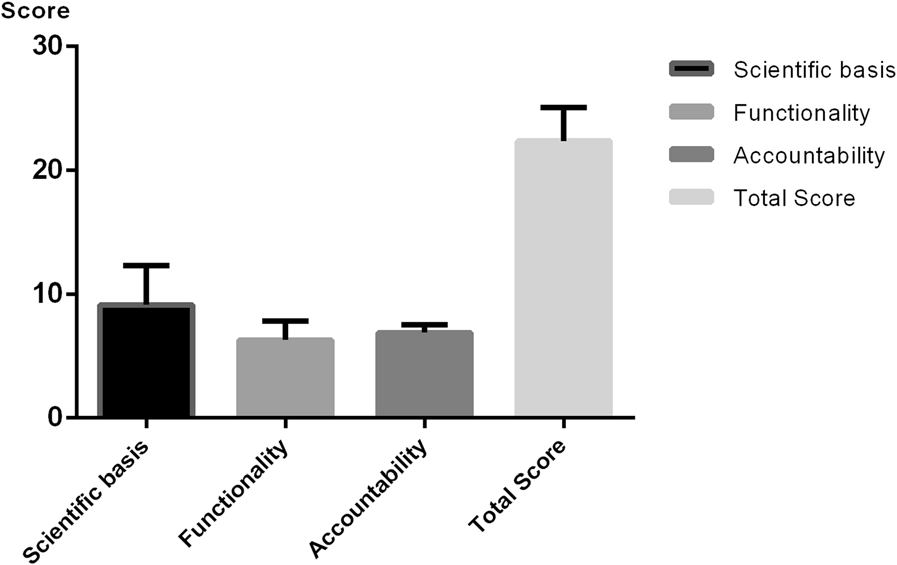
Average domain scores of apps with total scores of more than 20

For apps with a total score of more than 20, the average Scientific domain score was 9.15, the average Functionality and Usability score was 6.3, and the average Accountability score was 6.9 (Fig. 2).

#### Scientific basis

The mean Scientific Basis score was 5.51 ± 2.58 (out of 16). There were 2 apps with the highest score, (*Taiir SleepCare* and *Huadaifu*); both had scores of 14. Almost all of the apps provided actigraphy (86.61%). Only 3 apps claimed to have the capacity to record the electrical activity of the brain (electroencephalogram). No app mentioned anterior tibialis electromyography, chest or abdominal piezoelectric bands, chin electromyography, electrooculography, or nasal-oral airflow.

Approximately half of the apps were able to measure pulse (62.20%), and 1 app (*Mecare*) could perform electrocardiography. Some apps could record oxygen saturation (8.66%) by connecting to a pulse oximeter, which helped patients to recognize hypoxemia during sleep.

Eleven of the apps provided questionnaires to evaluate clinical symptoms; 8 of them used standard questionnaires, such as the ESS or BQ.

More than 80% of the apps provided reports of sleep structure (96.06%) and sleep stages (82.68%) based on actigraphy data. None of the apps provided AHI scores or REM/NREM stage sleep information.

#### Functionality and usability

The average Functionality and Usability score was 2.90 ± 1.84 (range, 0 to 8). The most common function among the apps was a smart alarm clock (44.09%), followed by a personal information record (39.37%). Consulting doctors (0.055%) and recording blood pressure (0.079%) were less common. The *Woniu Sleep* app had the highest functionality score. In addition to providing sleep surveillance, some apps could also record activity by counting steps and measuring distance (26.77%).

Ninety-eight apps required other equipment to monitor sleep conditions, and of these, 29 could also function independently. To analyze differences between those apps, we performed the Mann-Whitney U test. The results showed that in the Scientific Domain, the apps that used other equipment were superior to apps that worked independently (mean score 5.26 vs. 4.17, ***P*** < 0.001). This superiority was also demonstrated in the total scores of the apps (mean score 13.8 vs. 11.67, ***P*** < 0.001).

To analyze the correlation between Functionality and Usability and Scientific Basis, we performed Spearman correlation, and the correlation coefficient was 0.03 (***P*** = 0.746).

#### Accountability

The Silberg score of the 127 apps was 2.90 ± 1.84 (range, 2–8). Concerning authorship, the mean score was 2.71 out of 3. Almost all of the apps (125, 98.4%) provided the names of the authors. The mean disclosure score was 0.99. The score was very close to 1 because few apps provided information on sponsorship. The mean currency score was 1.84, and the mean attribution score was 0.26. The score for attribution was low because only 29 (22.83%) apps provided information regarding sources, and only 4 apps provided references.

A prior study found that few apps had a Silberg score ≥ 7 points [16]. In our study, there were 31 apps (24.41%) that had an accountability score ≥ 7 points. We divided apps into those with Silberg scores ≥7 and < 7 and used the Mann-Whitney U test to examine differences between the 2 groups. With respect to Scientific Basis and user rating, there were no significant differences between the 2 groups (mean score 6.26 vs. 5.27, *P* = 0.131; 3.87 vs. 3.64, *P* = 0.284, respectively). However, when the Accountability score was ≥7, the total score of the app was higher (17.48 vs. 13.16, *P* < 0.01), as was the Functionality and Usability score (4.03 vs. 2.53, *P* < 0.01).

The Spearman correlation coefficient between Scientific Basis and Accountability was 0.156 (*P* = 0.079) and that between Functionality and Usability and Accountability was 0.304 (*P* = 0.001).

#### Popularity

Of the apps, 76 (59.84%) had a popularity rating score, and 51 (40.16%) did not. The mean rating score for the 76 apps was 3.71 ± 1.06. The number of users who participated in rating the apps ranged from 1 to 8000 (mean ± standard deviation, 201.19 ± 913.31).

To analyze the correlation between user rating and the 3 domains of the evaluation scale, we used Spearman correlation analysis (Table 2). The user rating has the strongest relationship with Functionality and Usability. However, there were no relationships between user rating and Scientific Basis, Accountability, and total app score.

**Table 2:** The Correlation between Users Rating and three other domains

Spearman Correlation		Scientific basis	Functionality and Usability	Accountability	Total
Rating (*N* = 76)	Correlation Coefficient	0.034	0.317(**)	0.072	0.154
	*P* value	0.772	0.005	0.538	0.183

**: Correlation is significant at the 0.01 level (2-tailed)

#### Inter-rater reliability of evaluation

Two researchers analyzed the quality of these apps using the same evaluation scale. According to the previous study [17], we used the intraclass correlation coefficient (ICC) to analyze the inter reliability of the primary evaluation score and the final evaluation score. The ICC for the total score was 0.844 (*P* < 0.001), the ICC for scientific basis was 0.762 (*P* < 0.001), the ICC for functionality and usability was 0.715 (*P* < 0.001), and the ICC for accountability was 0.957 (*P* < 0.001). The scale we designed had relatively high reliability.

## Discussion

People in China have become more concerned about their health, and not only consult doctors but also surf the Internet to get information [18]. Mobile health apps play important roles in disease self-management and disease screening. Based on a Chinese survey in 2018, compared with nonusers, patients who used mobile health apps tended to have better short-term outcomes and better medical experiences [19]. Sleep apps are one of the most popular mobile health apps. In this study, we found high levels of interest and utilization of sleep apps in China, with the most popular app receiving more than 8000 comments.

Our content quality assessment showed that the total scores of most apps were low, especially in the Scientific domain. The maximum total score of our evaluation scale was 53, but the average score for all apps was only 14.23. Furthermore, the average score of scientific basis was less than one-fifth of the total score of the Scientific domain, indicating that the adherence of the content of these apps to the diagnostic criteria for OSA was low. Most apps only measured movement during sleep, which is not sufficient to diagnose OSA. In addition, movement recorded by the accelerometer in a mobile phone is questionable because of the low specificity. The *Sleep Time* app was one of the apps with total score of more than 20. However, a recent study showed that the *Sleep Time* app performed poorly when compared to PSG [20]. Thus, sleep parameters or sleep staging provided by mobile apps might be unreliable.

Some apps could connect to accessory devices, such as bracelets, bands and a micromovement-sensitive mattress with a sleep monitoring system. These apps provided more information.

In this study, some apps provided heart rate (62.20%) and oxygen saturation (8.66%) data by connecting to an oximeter. Other apps provided electrocardiogram and electroencephalogram data. No apps performed electrooculography or electromyography or measured nasal-oral airflow.

We analyzed the differences between apps that needed to be connected to other devices and the apps that could work independently. The results showed that when connected to other devices, the apps would be more scientific and reasonable. People can use these apps with accessory devices to monitor their sleep conveniently. In fact, the performance of accessory devices fluctuated greatly, especially in the measurements of sleep duration and sleep stage [21]. The correlation between accessory devices, such as an oximeter, and PSG is not high [22].

To improve sleep monitoring apps, researchers are working on optimizing algorithms, improving device design, and combining multiple devices to improve diagnostic efficiency. Portable monitoring (PM) has been suggested to shorten the time to diagnosis and to monitor the effects of OSA treatment [23–25]. In our study, some apps with a high Scientific score, such as the *Taiir SleepCare* app and the *Huadaifu* app, could be connected to portable monitoring devices.

We found that app popularity was significantly related to Functionality and Usability. People preferred multifunctional apps that could provide information about sleep, could play sleep-inducing music and could be used as a smart alarm clock to help people wake up at the best time. Some apps also provided the capacity to consult with a doctor. According to an online survey, this is the most popular function of mobile health apps [26]. However, Functionality and Usability was not correlated with the Scientific domain in our study. This suggests that apps with multiple functions were not necessarily better than other apps. Besides, there is no family doctor system in China, and doctors may not have time to provide prompt feedback online [27].

For web-based information, consumers and professionals usually use the Silberg score to judge the accountability of the information [15]. The mean Silberg score of our apps was 5.80 out of 9. This was lower than the Silberg score of depression-related websites, which was reported to be 6.47 out of 9 [28]. Most of the apps provided complete authorship information. Only 4 apps disclosed their sponsorship. The disclosure rate was much lower than the rate of 29.81% for obesity-management apps in Korea [16]. For attribution, only 22.83% of the apps provided information sources, and only 3.14% provided references, suggesting that the companies that designed these apps did not collaborate with reliable institutions. This might explain the low Scientific score of the sleep apps. For apps with higher Accountability scores, the Functionality and Usability score was generally higher. The Accountability score might reflect the quality of the designers. Professional designers would provide convenience for consumers, so the score of Functionality and Accountability would increase.

The adherence of app content to OSA screening recommendations was low. Considering the large number of app users in China, this should be recognized as a missed opportunity for OSA screening and the promotion of sleep quality. According to our study, apps should connect to reliable devices to collect sleep information, such as oral-nasal air flow, movement, and real sleep duration. Apps should also provide information about sleep, wake people up at the best time, and help them connect to doctors to obtain professional advice. An ideal app that can monitor sleep and screen for OSA should be designed by a collaboration between app designers and doctors. Studies have indicated increasing acceptance for remote sleep monitoring and screening for OSA [16, 29]. Some of the apps in our study could connect to portable monitoring devices, but we could not find user ratings for these apps. These apps may all be recommended by doctors, and consumers just regarded them as remote treatments rather than smart phone applications. People prefer multiple functional apps; thus, designers should optimize these apps to improve customers’ satisfaction.

At this time, most sleep apps are consumer apps, meaning that the main purpose of these apps is to earn money rather than to provide medical help. However, more and more people are downloading these apps to monitor their sleep. It is important to make sure that these apps can play a role in clinical surveillance or OSA screening. With the help of these sleep apps, sleep specialists could help more people who may suffer from OSA and could also monitor patients after diagnosis. Our results showed that great progress should be made to improve the quality of these apps to achieve these goals.

In our study, the ICC of the evaluation scale was quite high, meaning that the inter reliability of our evaluation scale was good. However, only two assessors conducted the evaluation procedure in our study, and the second assessor was not blind to the primary results; thus, more assessors should be recruited in the future to test the consistency of the evaluation scale.

### Limitations

While 127 apps were evaluated in this study, it is possible that some apps that met the inclusion criteria were missed. There is no published, validated, content analysis tool to evaluate sleep apps. Therefore, the evaluation scale for apps might not be comprehensive. In our study, the evaluation procedure was conducted by one assessor the first time, after which the results were verified by a second assessor. If there were differences between the two assessors, the results would be discussed by two assessors to obtain the final evaluation score. Therefore, the second assessor was not blind to the initial evaluation, which may have affected the accuracy of the results and the inter reliability. We did not recruit volunteers to test the consistency of Chinese sleep apps with PSG; this might be performed in the future.

## Conclusions

This study analyzed the quality of Chinese sleep apps using the content analysis method. The results showed the relative absence of Scientific Basis and Accountability among these apps. Apps that can connect to other devices were more scientific and powerful than others. Multifunctional apps were more popular and reliable. In the future, designers should cooperate with doctors and sleep specialists to design high-quality and multifunctional apps to support monitoring sleep and OSA screening.

## Supplementary information

**Additional file 1: Figure S1.** The Characteristics of Apps.

## Data Availability

All available data are provided in Additional file 1.

## Abbreviations

AHI: Apnea-hypopnea Index
Apps: Smart phone applications
ICC: Intraclass correlation coefficient
OSA: Obstructive sleep apnea
PSG: Polysomnography
REM/NREM: Rapid eye movement sleep/Non-rapid eye movement sleep
