# Supplementary material for: Quality analysis of smart phone sleep apps in China: can apps be used to conveniently screen for obstructive sleep apnea at home?
Source: BMC Med Inform Decis Mak. 2019 Nov 15;19:224. doi: 10.1186/s12911-019-0916-7 (PMC6858766; doi:10.1186/s12911-019-0916-7)
Supplement: Supplementary file 1 — Additional file 1: Figure S1. The Characteristics of Apps. [file 12911_2019_916_MOESM1_ESM.docx]

**Additional file 1: Figure S1. The Characteristics of Apps**

| Relative Rank | APP Name | Scientific Basis | Functionality and Usability | Accountability | Total Score | Number of Consumers Reviews | | User Rating |
| --- | --- | --- | --- | --- | --- | --- | --- | --- |
| 1 | Taiir SleepCare | 14 | 6 | 7 | 27 | 0 | \ | |
| 2 | UMindSleep | 13 | 6 | 7 | 26 | 1 | 5 | |
| 3 | Sleep as Android | 11 | 7 | 8 | 26 | 46 | 3.2 | |
| 4 | Huadaifu Sleep | 14 | 5 | 6 | 25 | 0 | \ | |
| 5 | Sleep Cycle alarm clock | 8 | 8 | 7 | 23 | 8000 | 4.6 | |
| 6 | Sleepace | 7 | 8 | 7 | 22 | 38 | 4.6 | |
| 7 | Beddit Sleep Monitor | 11 | 3 | 7 | 21 | 16 | 3.6 | |
| 8 | Sleep Time | 7 | 6 | 8 | 21 | 35 | 4.7 | |
| 9 | C life | 8 | 6 | 6 | 20 | 6 | 5 | |
| 10 | Xiaoyue Intelligence | 8 | 5 | 7 | 20 | 0 | \ | |
| 11 | Xiaoxi Health | 7 | 7 | 6 | 20 | 0 | 0 | |
| 12 | Auto Sleep | 7 | 6 | 7 | 20 | 828 | 4.6 | |
| 13 | Woniu Sleep | 4 | 9 | 7 | 20 | 4300 | 4.5 | |
| 14 | Mooring | 9 | 2 | 8 | 19 | 7 | 3.3 | |
| 15 | MLILY | 8 | 5 | 6 | 19 | 0 | \ | |
| 16 | Snoring Rabbit | 7 | 5 | 7 | 19 | 12 | 2.8 | |
| 17 | I Sleep | 11 | 1 | 6 | 18 | 3 | 2.3 | |
| 18 | Sleep Care | 10 | 1 | 7 | 18 | 0 | \ | |
| 19 | Fitsleep | 7 | 5 | 6 | 18 | 5 | 4.6 | |
| 20 | iwear | 7 | 5 | 6 | 18 | 10 | 3.3 | |
| 21 | Sleep Tracker | 7 | 4 | 7 | 18 | 5 | 3.8 | |
| 22 | UP | 6 | 6 | 6 | 18 | 61 | 4.4 | |
| 23 | Youning Sleep | 6 | 4 | 8 | 18 | 0 | \ | |
| 24 | Shuika | 7 | 4 | 6 | 17 | 0 |  | |
| 25 | BOE Sleep Health | 7 | 3 | 7 | 17 | 0 | \ | |
| 26 | Xiang Shui | 6 | 4 | 7 | 17 | 16 | 1.1 | |
| 27 | Pillow:Smart sleep tracking | 5 | 5 | 7 | 17 | 1400 | 4.7 | |
| 28 | Anshui Elf | 8 | 4 | 4 | 16 | 0 | \ | |
| 29 | Mecare | 8 | 2 | 6 | 16 | 5 | 4 | |
| 30 | Likang Medicine Assistant | 8 | 2 | 6 | 16 | 0 | \ | |
| 31 | Zhongyi Sleep Manage | 8 | 2 | 6 | 16 | 0 | 3 | |
| 32 | Mengjia | 6 | 5 | 5 | 16 | 0 | \ | |
| 33 | Hero Band | 5 | 5 | 6 | 16 | 70 | 4.3 | |
| 34 | AHB sleep tracker | 4 | 6 | 6 | 16 | 0 | \ | |
| 35 | HELPandGIVE | 8 | 2 | 5 | 15 | 1 | 5 | |
| 36 | Xianjue | 8 | 1 | 6 | 15 | 0 | \ | |
| 37 | Shendeng Sleep Keeper | 8 | 1 | 6 | 15 | 0 | 3 | |
| 38 | Xiaomi Sports | 7 | 2 | 6 | 15 | 2700 | 2.9 | |
| 39 | Shuimian Guanjia | 7 | 2 | 6 | 15 | 0 | \ | |
| 40 | Misfit | 6 | 3 | 6 | 15 | 3800 | 4.8 | |
| 41 | Keshuichong | 6 | 3 | 6 | 15 | 47 | 3.6 | |
| 42 | Nuoan Sleep Companion | 6 | 3 | 6 | 15 | 0 | \ | |
| 43 | CSleep | 6 | 3 | 6 | 15 | 0 | \ | |
| 44 | nokia health mate | 6 | 3 | 6 | 15 | 7 | 2.65 | |
| 45 | Sleep Master | 6 | 2 | 7 | 15 | 1 | 5 | |
| 46 | Beijiashui | 6 | 2 | 7 | 15 | 0 | \ | |
| 47 | Cling | 5 | 3 | 7 | 15 | 27 | 2.9 | |
| 48 | i-gotU Life | 5 | 3 | 7 | 15 | 2 | 1 | |
| 49 | Yinghuochong Sleep | 4 | 5 | 6 | 15 | 1430 | 4.8 | |
| 50 | i-Pillow | 4 | 5 | 6 | 15 | 0 | \ | |
| 51 | Yixiu Sleep | 2 | 5 | 8 | 15 | 11 | 3.3 | |
| 52 | Snoring Oxigen Ring | 9 | 0 | 5 | 14 | 0 | \ | |
| 53 | SuperSleep | 8 | 0 | 6 | 14 | 0 | \ | |
| 54 | Zhishuibao | 7 | 2 | 5 | 14 | 0 | \ | |
| 55 | Good Night | 7 | 1 | 6 | 14 | 0 | \ | |
| 56 | Tiantian Bracelet | 7 | 1 | 6 | 14 | 54 | 2.05 | |
| 57 | Happy Sleep | 7 | 1 | 6 | 14 | 2 | 5 | |
| 58 | YLsmart | 5 | 4 | 5 | 14 | 3 | 4.3 | |
| 59 | Fitband | 5 | 3 | 6 | 14 | 6 | 3.5 | |
| 60 | JoyFit K2 | 5 | 3 | 6 | 14 | 0 | \ | |
| 61 | Quick Sleep | 5 | 3 | 6 | 14 | 6 | 5 | |
| 62 | NT Sleep | 5 | 1 | 8 | 14 | 0 | \ | |
| 63 | Beiwo sleep | 4 | 4 | 6 | 14 | 11 | 3.7 | |
| 64 | Good Sleep | 4 | 4 | 6 | 14 | 0 | 5 | |
| 65 | Sleep Scientific Alarm Clock | 4 | 4 | 6 | 14 | 11 | 4.8 | |
| 66 | Smart Alarm Clock | 4 | 4 | 6 | 14 | 10 | 3.2 | |
| 67 | EasyFit+ | 4 | 4 | 6 | 14 | 0 | \ | |
| 68 | SleepBot | 4 | 4 | 6 | 14 | 35 | 3.95 | |
| 69 | Kaola Sleep | 4 | 3 | 7 | 14 | 148 | 4.9 | |
| 70 | Morning Alarm Clock | 4 | 3 | 7 | 14 | 435 | 4.5 | |
| 71 | TPF Sleep keeper | 9 | 0 | 4 | 13 | 3 | \ | |
| 72 | LELOXE | 7 | 0 | 6 | 13 | 0 | 3 | |
| 73 | Zhihandian | 6 | 2 | 5 | 13 | 0 | \ | |
| 74 | YomoSleep | 6 | 2 | 5 | 13 | 0 | \ | |
| 75 | Zhima Sleep | 5 | 3 | 5 | 13 | 0 | \ | |
| 76 | Sleep Care | 5 | 3 | 5 | 13 | 0 |  | |
| 77 | Polar Flow | 5 | 2 | 6 | 13 | 702 | 4.8 | |
| 78 | Feixun Sports | 5 | 2 | 6 | 13 | 58 | 2.7 | |
| 79 | Meizu Bracelet | 5 | 2 | 6 | 13 | 73 | 2.6 | |
| 80 | Actino Link for WB004 | 5 | 2 | 6 | 13 | 0 | \ | |
| 81 | EasyFit | 5 | 2 | 6 | 13 | 0 | \ | |
| 82 | Mengjie Sleep | 4 | 5 | 4 | 13 | 3 | 5 | |
| 83 | Comfortable Time | 4 | 5 | 4 | 13 | 32 | 3.65 | |
| 84 | airweave sleep analysis | 4 | 4 | 5 | 13 | 0 | \ | |
| 85 | iSleep | 4 | 2 | 7 | 13 | 0 | \ | |
| 86 | Sleep Recorder | 2 | 4 | 7 | 13 | 6 | 4.5 | |
| 87 | Sleep Q | 7 | 0 | 5 | 12 | 0 | \ | |
| 88 | Gloden Sleep | 6 | 0 | 6 | 12 | 0 | \ | |
| 89 | Sleep Tailor | 6 | 0 | 6 | 12 | 0 | \ | |
| 90 | Health Bracelet | 5 | 3 | 4 | 12 | 0 | \ | |
| 91 | Keyband | 5 | 1 | 6 | 12 | 12 | 3.25 | |
| 92 | Wanke | 5 | 1 | 6 | 12 | 1 | 5 | |
| 93 | Runtastic sleep better | 4 | 4 | 4 | 12 | 824 | 4.1 | |
| 94 | 37° Bracelet | 4 | 2 | 6 | 12 | 11 | 2.8 | |
| 95 | GE Fitness | 4 | 2 | 6 | 12 | 0 | \ | |
| 96 | Qianli Health | 3 | 3 | 6 | 12 | 6 | 4.8 | |
| 97 | iCreBand | 3 | 3 | 6 | 12 | 3 | 2.65 | |
| 98 | SnoreLab | 1 | 4 | 7 | 12 | 150 | 4.7 | |
| 99 | Maiwei Sleep Manage | 9 | 0 | 2 | 11 | 0 | 3 | |
| 100 | Tianmu Intelligence Pillow | 8 | 0 | 3 | 11 | 0 | \ | |
| 101 | Sleep Surveillance | 5 | 3 | 3 | 11 | 5 | 4 | |
| 102 | BleStep | 5 | 3 | 3 | 11 | 0 | \ | |
| 103 | Kanglehui | 5 | 2 | 4 | 11 | 134 | 5 | |
| 104 | Hej Band | 5 | 2 | 4 | 11 | 0 | \ | |
| 105 | Haohao Sleep | 4 | 2 | 5 | 11 | 0 | \ | |
| 106 | Shuilema | 4 | 2 | 5 | 11 | 0 | \ | |
| 107 | HeHa | 4 | 1 | 6 | 11 | 249 | 4 | |
| 108 | DreamZ - Lucid Dreaming. Control your dreams! | 4 | 1 | 6 | 11 | 0 | \ | |
| 109 | PAPA Bracelet | 3 | 2 | 6 | 11 | 6 | 3.5 | |
| 110 | MooFit | 3 | 2 | 6 | 11 | 8 | 4 | |
| 111 | Sleep Timer | 2 | 2 | 7 | 11 | 0 | \ | |
| 112 | GX_Smart | 5 | 2 | 3 | 10 | 0 | \ | |
| 113 | iHappySleep | 3 | 1 | 6 | 10 | 1 | 3.6 | |
| 114 | Qingxiang+ | 2 | 3 | 5 | 10 | 2 | 4 | |
| 115 | Shuashua Bracelet | 2 | 2 | 6 | 10 | 35 | 1.9 | |
| 116 | Shumianle | 4 | 0 | 5 | 9 | 0 | \ | |
| 117 | SmartWBA | 2 | 2 | 5 | 9 | 0 | \ | |
| 118 | Baojing Sleep Analysis Meter | 2 | 2 | 5 | 9 | 1 | 3.15 | |
| 119 | Sleepstats | 1 | 2 | 6 | 9 | 0 | 3.6 | |
| 120 | Sleep Diary | 1 | 2 | 6 | 9 | 2 | 3.15 | |
| 121 | Snoring U | 1 | 1 | 7 | 9 | 0 | \ | |
| 122 | Earphone Record Sleep | 4 | 2 | 2 | 8 | 0 | 3 | |
| 123 | Sleep Snoring Test | 1 | 2 | 5 | 8 | 0 | 3 | |
| 124 | Sleepmeter Free | 1 | 1 | 6 | 8 | 0 | 3 | |
| 125 | GoSleep | 4 | 0 | 3 | 7 | 0 | \ | |
| 126 | Sleep Analysis Meter | 1 | 2 | 3 | 6 | 0 | 3 | |
| 127 | Sleep Course Recorder | 0 | 2 | 4 | 6 | 1 | 3.15 | |

**Relative ranking**: Ranking was determined according to the total score of apps. For the apps with the same total score, we sorted them according to the scientific basis score.

**App name**: For apps without English name, we used Chinese Pinyin to describe the apps.
